# An Overview of the Sociological and Environmental Factors Influencing Eating Food Behavior in Canada

**DOI:** 10.3389/fnut.2020.00077

**Published:** 2020-06-03

**Authors:** Massimo F. Marcone, Pavneesh Madan, Bernard Grodzinski

**Affiliations:** ^1^Department of Food Science, University of Guelph, Guelph, ON, Canada; ^2^Ontario Veterinary College, University of Guelph, Guelph, ON, Canada; ^3^Plant Agriculture, University of Guelph, Guelph, ON, Canada

**Keywords:** food security, food policies, eating behavior, Health Canada, biotechnology

## Abstract

This review extensively discusses various socio environmental factors affecting eating behavior of the general public within Canada including the development and implementation of national policies. A framework representing the determinants of healthy eating can be grouped into four categories i.e., the individual determinants, the economic environment, the social environment and the physical environment. This framework allowed for addressing food insecurity and social economic ecosystem of Canadians. Lastly, we investigate the role in which biotechnology plays in improving food security and addresses the significant impact biotechnology has contributed toward on agriculture and the food market. Overall, this review using such sources as Web of Science, Pub Med and Scopus provides significant contribution toward understanding the social economic environment and eating behavior of people living in Canada. In conclusion, this has led to identify a research gap as there is a significant need to address the development and implementation of policies in the food and nutrition environment.

## Introduction

**Figure 1:**
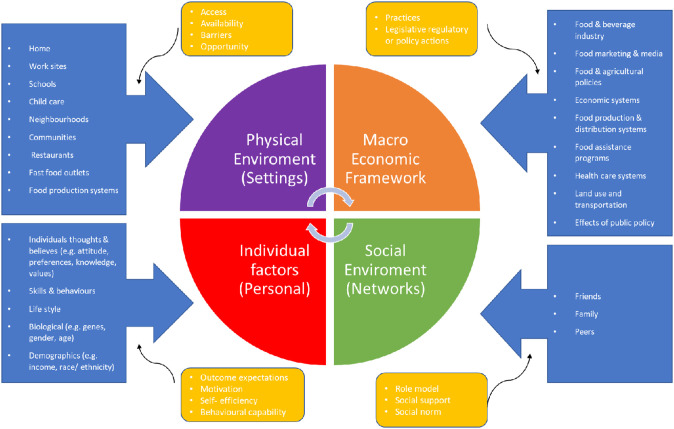
A systematic representation of the four determinants that affect an individual's eating behavior.

The eating behavior for an individual is a highly personal and complex process involving the interplay of multiple factors that impact one's health and nutrition. These factors can include the individual's thoughts and beliefs, their close social environment that includes (like) family and friends, their physical environment at (including) home and workplace, and the policies which govern the society they live in (just to name a few). A personal ecological framework comes in handy to conduct research, intervention, policies related to feeding habits and social economic activities including focusing on the linkage between people and their environments (1–3). A framework representing the determining factors of good feeding habits for an individual can be divided into four major groups (Figure 1). These include (1) Personal determining factors (2) State of the economy, (3) the social landscape, and (4) the ecological environment in which the individual resides (2–4). Personal factors include characteristics and behavioral factors that affect an individual's choice of food (physiological state, food preferences, nutritional knowledge, perceptions of healthy eating and psychological factors) (3). Categorized in the second group is the economic ecosystem which includes prices of food, food security, income, and employment (5). The third group, the social environment, includes people with whom an individual relates with on a daily basis including culture, family, acquaintances, friends, and social media (6). Finally, the physical environment encompasses all infrastructures that affect food availability such as food outlets in their environment including work sites, schools, restaurants, and supermarkets (7).

In this review, it is the other three (3) factors (governing the ecosystem) other than the individual factors that affect the feeding habits as well as the effects of public policies on the population as a whole that will be discussed extensively.

## The Economic Ecosystem

### Problem of Food Security in Canada

According to the United Nation's Food and Agriculture Organization (FAO) food security can be defined as “a situation whereby safe, nutritious and sufficient food is available and easily accessible to all members of a given population which allows them to meet their required nutritional needs in other to live an active and healthy life without losing their dignity” in a fashion that maintains human dignity in our society (8). The feeding habits of a family depend heavily on the total income made at month end. Low incomes more likely result into poor feeding habits and the food security of individuals (9). Low house-hold income triggers many health inequalities within Canada. In general, population groups with the highest poverty rates are reported to undergo the worst health status, including nutritional health, obesity and development of chronic diseases (10). Reports released in 2004 revealed that approximately 9 out of every 100 Canadians experience food insecurity or “food deserts” (an urban area where it may be difficult to buy good quality fresh food which is affordable) at sometimes during the year (11). This would indicate that about 2.7 million Canadians, which includes 777, 200 children suffer from food insecurity. More recently, in 2018 statistics published by Household Food Security Survey Module (HFSSM) and Canadian Community Health Survey (CCHS) revealed that there is a huge difference between the availability and cost of healthy food across different neighborhoods in Canada (12). They reported that ~1 household in every 8 in Canada have food insecurity or insecure access to food; this figure is expected to amount to over 4 million Canadians including 1.15 million children. Studies have shown that food insecure households lead to poorer physical and mental health. For instance, a study performed by Jessiman-Perreault and McIntyre (13), reported the likelihood of individuals suffering from food insecurity to also suffer from poor mental health. The authors reported that the severity of food insecurity increases the probability of the affected individual going into depression, becoming over anxious, mood swings and also thinking of suicide. The recognition that food insecurity affects mental health and performance was further buttressed by the findings of Davison et al. (14) and Muldoon et al. (15).

Several factors have been indicted as the cause of hunger and food security in Canada and some of these factors include /s/ loss of job, high rate of unemployment, homelessness, drug abuse, high cost of medical services, unavailable or unaffordable food in one's surroundings (16). The categories of people who are most exposed to food insecurity are the poor, new immigrants, the under employed, the homeless, and senior citizens (16). These groups are not exclusive, however, as a well-educated individual or family can also experience food insecurity. It is important to understand that education has little or no effect in cushioning the effects of food insecurity (17). If food is expensive or inaccessible, even a person who has a degree in nutrition and healthy eating would suffer the same condition as people who have no knowledge of the course at all.

Sadly, people have resorted to food banks as their go-to-option in order to find a solution to problems of hunger and food insecurity in Canada. These food banks are voluntary, extra-governmental programs or Non-Governmental Organizations NGO's that advocate for support from all sectors of society to provide food for hungry individuals and families. Generally, running a food bank relies heavily on donations and also volunteers who are willing to give their services for little or no financial rewards (18). Several studies have revealed that many of the donated foods often fall short of nutritional standards for an adult. This is due to the fact that the foods received are primarily nonperishable food that can be stored for a long period of time. Perishable foods such as dairy, meat and fresh products may be unavailable thereby leaving the food banks with stocks of foods that provide less health benefits (19).

Food insecurity has become too common in many indigenous communities where earning an income is limited. Another factor that increases food insecurity is the high cost of transportation to rural communities. Communities that are in the extreme end of the map have little or no access to fresh food, dairy products, and leaner meats (19). In addition, shop owners and food dealers serving these areas often prefer to stock nonperishable foods rather than the perishable ones (19). In order to combat this problem, communities living in remote areas have devised some means which include reserves, community freezers, and community sharing practices (19). For example, in recent years, the establishment of a community freezers in such places as Nain, Newfoundland and Labrador are helping families in regions that struggle with affordable food while building on Inuit traditions of sharing food. These freezers may be stocked with wild meat like seal meat, Arctic Char and moose, free of charge to anyone in need. Currently, there is an increased interest in developing controlled environment production of key horticultural products but such effects though increasing are not advanced in any but a few communities.

The effects of food insecurity may not be evident in all members of suffering families because often times parents, mothers especially, would do everything possible to ensure that their children are well fed even if it means that they go hungry. Therefore, in order to ascertain if a family is suffering from food insecurity, the quality and quantity of food available to the mother needs to be known (17). Mothers that eat less nutritious food may lead to an impact on breast milk, which ultimately affects an infant's development. In fact, B vitamins such as thiamin, riboflavin, B6 and B12 as well as vitamins A and C found in breast milk are influenced by the mother's diet.

Often times, people suffering from obesity are thought to have access to good food, but it is rather unfortunate to realize that obesity is common among the less affluent citizens. This condition is described as the OBESITY PARADOX and it is due to the fact that in order to avoid being hungry, this set of people eat lower priced-energy dense products which are largely advertised and highly convenient because of extended shelf-life and readiness to prepare (20).

Achieving food security specifically in low income households, is dependent on improving access to food which is not only high in essential nutrients but equally safe and affordable. Canadians require well-planned and lasting measures to relieve food insecurity within low income households. Improving food security in Canada should consider the importance of supply and demand chain in creating a solution to food security. All stake holders should be informed, and it should be ensured that they agree and comply with the legislation.

### The Prices of Food in Canada

Another, important factor affecting food insecurity in Canada and globally is the cost of food especially in low income households which leads to excessive calorie intake which can be an obesogenic factor leading to insufficient foods (due to “food deserts”). However, Canada can boast of having one of the cheapest food markets among developed as revealed in reports published in 2008 which state that Canadians spent just a 10th of their salaries on food (21). By 2016, this has risen to over 14% and is believed to grow at even a faster rate within the next few years (22). Many affluent Canadians accumulate more calories than their body require because food is relatively cheap and also the sedentary nature of their job require little or no physical activity. With the modern era changes to a more automated society, factories/workers exert less energy and those who work behind a computer now possess more sedimentary positions compared to the past. Report shows that there has been an increase in the poor feeding habits of these categories of people because they barely have time to prepare meals at home but rather depend on restaurants daily for their meals. Other factors contributing to the poor feeding habit among the affluent one may include cheap food, double-income work schedules, and the increase in the number of restaurants and prepared food outlets (11). Based on the Canada Food Guide (23), literature reports have shown that many low-income households are able to easily afford healthy foods. Fast foods and junk foods are quite affordable than organic foods such as milk, meat, grains and veggies. There may be huge problem for public policies if it confirmed that it is easier and cheaper to buy unhealthy foods than to buy healthy ones. Pricing greatly affects the choice and consumption of healthy foods (including fresh fruits and vegetable and those foods having good quality proteins from diary and animal sources), therefore understanding the impact of social economic factors is already a first step toward determine the effectiveness of a community-based, food pricing policies that can improve feeding habits of Canadians.

## Social Factors Affecting Healthy Eating

Food choice and the eating habits of an individual in Canada are highly variable and largely influenced by many interrelated factors, amongst them include social and cultural factors (2, 4). The choices of food made by individuals can be influenced by his/ her cultural upbringing and the type of company he/she keeps would also sometimes dictate his/her interest and opinion about some food types (24). This section discusses the effects of social and cultural factors affecting the eating habits of an individual.

### Family Network

Children's dietary patterns evolve within the context of the family. The way of life of a given family affects the eating habits of its children because provision of certain foods often times determines the eating practices of these children (2). A strong correlation has been reported to exist between the feeding habits of parents (mothers in most cases) and those of their children (2). When parents show positive attitude toward healthy food during meals, especially when it's a new delicacy that their children are eating for the first time, it can make meals more enjoyable. Healthier foods have been found to be associated with family meals. This includes more vegetables, fruits, grain products, calcium-rich foods (2). The frequency of family meals has been shown to relate to the nutritional well-being of adolescents and children. In fact children who share more than three family meals per week are more likely to be of normal body weight, have healthier eating and dietary patterns than those who do not (25). It has also been shown that children in single parent families, reconstituted families, or without parents are more likely to have unhealthy eating habits (26). Interestingly, the presence of grandparents simultaneously prioritize healthy foods and may help in feeding while at the same time introduce food treats which are usually found to be unhealthy (27).

How a family is set up generally shapes the level of exposure children have to new delicacies, rules of mealtimes and healthy food practices (2). Authoritative parenting styles where children are not allowed to eat less healthy foods regularly, but parents offer it to them as rewards for good deeds have proven to increase the desire in children for poor foods (2). On the other hand, some parents may unconsciously encourage poor feeding habits in their children by allowing them take foods with high calorie level as snacks (2). Parents must learn to strike a balance between the two cases of permissive and authoritative parenting (28).

### School Environment

The school environment also plays a big role in influencing children's and adolescents' eating habits and choices. Academic environment have been indicted as a place conducive for kids and teeneagers to learn and inculcate healthy feeding (4). As children begin schooling, they start to meet with several other children who can influence either positively or negatively their feeding habits. This peer influence becomes stronger as these children begin to grow into adolescence, and they are away from their parents preying eyes. On a daily basis, Children and adolescents takes (consume??) at least one meal and a several snacks without the supervision of their parents because of the amount of time spent in school (29). Research shows that kids that are well-fed usually score better grades and are also behaviorally and emotionally sound (29). Therefore, schools should not only provide healthy meals through federally reimbursable school nutrition programs but play a vital role in providing enabling environments for students to inculcate healthy eating habits and encourage the intake of good foods (29, 30). In fact, nutritional education curriculums that develop to inspire new more healthy dietary habits amongst children, by using garden-based interventions have had promising results (31).

### Social Support and Seniors

The older individuals become, the weaker their sight, voice and olfactory senses leading to choices of foods solely depends on the strength of their teeth. Additionally, the aging population (60 years and above) are more vulnerable to developing diseases such as cardiovascular, metabolic syndrome, osteoporosis, and cancers which ultimately affect the overall health of senior people (32). These factors can have negative effects on the appetite and food consumption of an older person (33). Isolation and loneliness may also lead to the individuals losing interest in eating which may expose them to malnutrition. Programs like Meals on Wheels© have assisted aged citizens who live in their personal homes to alleviate loneliness and also provide ready-made balanced meals for them (33). Seniors who often are on reduced incomes and might struggle to buy healthy foods and this might lead to malnutrition and health challenges. Older people who reside in a social environment, pay proper attention to their diet, learned, stops smoking, and exercise regularly have been found to have a better nutrition (33).

### Culture

Culture simply means the way a group of people live their lives. It is the sum total of “the values, beliefs, attitudes, and practices accepted by members of a given community whether ethnic, religious, or social” (34). Culture can strongly influence the feeding habit of individual or a group of people and in a manner that is not unconnected to the fact that different cultures have foods and cuisine peculiar to them (35). Culture may determine the way people eat and what their beliefs are as regards to food (36). Often during community celebrations, eating is usually done with others which give individuals a feeling of acceptance. The myths of cultural and prevalent multicultural history and activities in prominent areas of Canada creates memories that appeals to eating and food and feeding culture of participants. There are a number of difficulties faced by people from ethnic minorities; they may face ethnic marginalization because of what they have decided to believe and how they have chosen to live their lives due to their cultural back ground and this might prevent them access food as they do not have good paying jobs and they also reside among people of lower socioeconomic status (35). They may also find it difficult to have access to traditional foods therefore left with no other choice than to feed on foods that they are not used to eating. For people who are not native English speakers, it might be difficult for them to understand food labels or even locate where to shop. This having been said people from remote and traditional cultures sometimes may have remarkable resiliencies on food systems due to their rich culture of cultivating and conserving than those in cities or urban areas.

### The Influence of the Mass Media

Food marketing through the media is one of the multiple factors influence the eating behavior of people and many youth. Over the past decade, Canadian kids and teenagers have been the main target for intense advertisement for food which ranges from messages we see everywhere ranging from watching television (TV), listening to the radio, reading newspapers and magazines even using social media (37, 38). Advertisements and product promotions have successfully influenced the choice people make regarding foods, beverages, restaurants, and cafeterias. Recently, Kaiser Family Foundation performed the biggest research study on the impact of food advertisements on television stations on children (age of 8–12) (37). They reported that children see at least 21 adverts daily and about 7,600 yearly, all geared toward promoting chocolates, sweets, and all kinds of sugary snacks with none of the 8,854 ads reviewed selling fruits and vegetables. The sort of products being pitched to these kids have been shown to be high sugar, fat, and calories and are generally unhealthy. Studies have shown there is a probable correlation between TV advertisements and the growing obesity epidemic (39, 40). Considering that obesity is one of the biggest public health problems in this century, more research studies need to shift its focus on evaluating factors affecting childhood obesity with regards to mass media advertisement. Additionally, policies should be implemented to provide recommendations for the food, beverage, and restaurant industries to focus their attention on promoting and advocating food that are healthy and help children and young adults attain good health.

## Effects of the Physical Environment on Healthy Eating

The degree to which society is lured into making poor feeding decisions is heavily dependent on several aspects such as the immediate environment (food availability and accessibility in the environment) and internal factors (e.g., food preferences). The physical environment includes all physical structures, geographical areas, and factors (built environment) around us that determines food availability and accessibility (2). These factors includes all structures designed by man which includes road networks and buildings which gives rise to neighborhoods and transportation networks, distance to grocery stores (whether walking is possible or other means of transportation is required), closeness to fast-food outlets, and availability of homemade among many other factors (35). Both centralized and decentralized food production and distribution systems have their inherent vulnerabilities.

The evaluation of the physical environment is often based on its involvement in promoting or hindering healthy feeding habits and food security. According to recent research, it was uncovered that lower income neighborhoods and seniors' facilities do not have access to so many options as regards foods and groceries when compared to the wealthy neighborhood where there are several stores and convenience with a wide range of products from which to choose (35). People who live in the low-income neighborhood have to rely on the little options available to them which may not have the required nutrient contents and sometimes expensive (2). In contrast, the high earning citizens have access to big grocery stores with a wide range of affordable foods to choose from, and lesser number of fast-food outlets.

## The Interaction of Determinants on the Obesogenic Environment

The Obesogenic Environment refers to an environment that allows and encourages its inhabitants to take consume a lot of energy (overeating, unhealthy eating) and expends little or no energy (lack of physical activity) (37, 39). Therefore, an individual who consumes food with high calorie value but performs little or no physical activities predisposes him/her to becoming overweight (37, 40). This can ultimately cause an obesity epidemic (37, 40). The length at which people get lured into making unhealthy feeding decisions depends on the several factors. For instance, the development of genetically modified foods has made fast-food available and affordable, but these foods contain high amounts of fat, sugar, sodium, and calories but little amount of the essential micronutrients (37, 40). Portion size also plays a crucial role in the obesogenic environment and affects the number of calories consumed with portions of energy-dense foods in Canada becoming steadily larger (41). In recent decades, portion sizes of family style and fast-food restaurants have been on the rise, people being offered larger portions which increases their caloric intake. Therefore, consumers are lured into consuming bigger portions of food because the prices are low and everyone wants to maximize the value they receive from their money (42, 43). Another factor includes time and availability as families whom both parents have paid employment have little or no time cook at home therefore the easiest way out to patronize fast food restaurants and pre-made foods vendors (42, 43). The improvement in the quality of advertisement and the advent of social media marketing has greatly increased the awareness and cravings for less healthy food among Canadians (44). The suburbia was designed such that for you to reach anywhere, you must make use of vehicles and sidewalks with bike paths not always accessible or available (45). All of the following factors; economic, social, and environmental contribute to the creation of our “Obesogenic Environment.”

## Policies and its Role in Promoting a Healthy Food Environment

Canada has witnessed a sharp increase in the number of overweight, obesity and diet related non communicable diseases (NCDs) across several facets and class of people and most importantly amongst vulnerable populations(such as indigenous populations and those with low socioeconomic status) (46). There seems to be an important opportunity to take steps to combat food and nutrition environment. But what is a policy? Simply defined, a policy is the strategic way or method adopted by a company, individual or government to tackle a particular situation (47). In food and nutrition, government is the sole decider of policies in order to foster a healthy social and physical community (47). For instance, the federal government put together the Eating Well with Canada's Food Guide to cater for the healthy and balanced feeding of Canadians from the tender age of 2 (48). At the Federal level Health Canada, a government parastatal, promotes and supports the nutritional health of Canadians. Health Canada provides several functions, with one being developing, promoting and implementing evidence-based policies for food and nutrition (49). The Canadian Food Inspection Agency is the custodian of the policies.

Health Canada is responsible for new policies and then expect players in the industries to comply without it being enforced on them. For instance, McDonalds was the first to act accordingly when Health Canada proposed that trans-fat be banned (50). Health Canada applied this same approach when it proposed reducing sodium contents in the diets of Canadians (51). Although the government has the power to control several aspects of food packaging and labeling, however, there is little or nothing it can do to control advertisement. It is known that advertisements and logos influence the eating behavior of the general public. As described within this review, advertisements tend to mainly promote food and beverages that have lower nutrient content but have higher fat, sodium, and sugar contents; comparatively there are only few ad that are made for fruits and vegetables (47). These advertisements mainly target kids and teenagers, both groups who are vulnerable (3). Therefore, much debate has been centered on whether government should develop policies toward regulating or setting standards or basic guidelines for the food and beverage industry to adapt and implement (3). There is an ongoing study by Health Canada to determine criteria for defining “healthy” and “less healthy” foods and beverages might have positive impacts in this case (51). If the study returns a positive result, then there is light at the end of the tunnel for population-wide healthy eating. Food policies can also be applied to the areas such as schools in order to create enabling environments for healthy feeding habits. Several provinces have put in place legislations that guide the choice of foods and beverages that may be sold in schools. Several health practitioners believe that for the policy to be effective, the government needs to extend it to secondary schools and should cover every form of food sold to students (52). Some districts have policies that govern foods in some secondary school cafeteria and public events like fund raising, (e.g., replacing chocolate with grapefruit at fund raisers), and special events (e.g., pizza and hot dog days). Importantly, regulations and interventions geared toward improving the food environment should move this responsibility away from individuals to a collective behavior that either encourages or discourages the choice of good foods (52).

### Calorie Labeling on Menus

About a million of Canadians suffer from Obesity which has been declared as an important public health challenge (53). According to the 2015 Canadian Health Measures Survey, ~5.3 million adults are obese (54, 55). Which is equivalent to roughly 28.1% or more than one in four adults in Canada are obese and may need the help of medical professionals to leave with the disease (56). Recently, obesity has been declared to be a serious source of health concern for the Canadian health care system with total cost directly related to obesity (including physician, hospitalization and medication costs) exceeding $7.1 billion annually (56).

As Canadians consume more fast foods and eat more unhealthy meals outdoor, the vast majority of the population battles with keeping their maintaining a healthy body weight. Health practitioners have put up suggestions that will require restaurants to make nutrition information available to customers just as food manufacturers does on their product packages (56). Research reveals that many people, including health practitioners, find it difficult to determine the level of calorie present in a meal served in restaurants (57). In a joint effort by both the Ontario Provincial Government and the government of British Columbia, legislative actions may be taken against restaurants that refuse to comply with the policy of stating the nutritional information of their menu (58). Similarly, one strategy or policy that the United States used to combat the increasing prevalence of overweight and obesity was to ensure that nutritional labels were placed on menus of restaurants and fast food outlets (59).

### Nutrition Labeling on Packaged Foods

*Eating Well with Canada‘s Food Guide (released in 2017)* among several other programs has been put in place by Health Canada in order to inform the general public on the benefits of healthy eating.

In 2004, the Canadian government developed and implemented a strategy to inform consumers about the number of calories and other nutritional content in all packaged foods. The strategy states that for every product released into the market, the manufacturer must include a Nutrition Facts Panel on the package. The nutritional labeling information is standardized and strictly regulated by the Nutrition Facts Panel and should contain the following labeling information. Information that must be included: a list of ingredients used in production, the calorie content and the amount of other ingredients per serving. The ingredient list has always been included on the package and it is still retained in order to inform consumers about ingredients that may have allergic reactions so that they can avoid the food all together. The percent Daily Value (% DV) column provides information on the amount of nutrients requirements that a person takes in per one serving of the food; this is based on a 2,000-calorie diet (70) which estimates amount of calorie required by a the energy or caloric requirements of immobile woman capable of giving birth to children.

In 2004, Health Canada permitted and endorsed those involved in food business to advertise certain health benefits associated with their goods. Only two kinds of proofs were permitted, namely health and nutritional claims. Health claims are proclamations that rely on the facts proven scientifically that some health benefits can be gained from specific product. The Canadian Food Inspection Agency is very strict with the wording of each permitted statements. Six health claims are currently allowed to be used in Canada: “a healthy diet low in sodium and high in potassium and reduced risk of high blood pressure”; “a healthy diet with adequate calcium and vitamin D and reduced risk of osteoporosis”; “a healthy diet low in saturated and trans-fat and reduced risk of heart disease”; “a healthy diet rich in vegetables and fruit and reduced risk of some types of cancers”; “non-fermentable carbohydrates in gums and hard candies and reduction in dental caries”; and, the newest one, “Plant sterols help reduce [or help lower] cholesterol” (60).

Nutritional claims are statements about the quantity of a particular nutrient a food may or may not contain. For example, “source of fiber” or “low in saturated fats” are both permitted nutrient claims. These claims are on a gradient and the wording is regulated. For example, a “source of fiber” claim means that for every serving of the product, you'll receive at least 2 g of fiber whereas a “high source of fiber” means you will receive 4 g of fiber in that serving, and a “very high source of fiber” claim means a serving will provide at least 6 g of fiber (61).

The demand from consumers for healthier and alternative products has spurred the food and beverage industry to promote and reformulate products in order to meet the demands of consumers. In order to promote these new reformulated products and in bid to assure consumers of the safety of their products, manufacturers have devised a plan to print logos or symbols on their products (62). The idea is not regulated by the government; therefore, the manufacturers has the autonomy to decide the criteria to pass any product as safe (62). A good example of the third-parties (or independent) logo programs in Canada is the Heart and Stroke Foundation‘s *Health Check*™ (http://www.healthcheck.org/). For a product to be accepted as safe and make use of the *Health Check*™ logo, the product must contain the standard amount of saturated fat, sodium, and sugar. Also, manufacturers will be required to pay a token to have their products tested. Health Canada has planned to make adjustments to the table that gives information about the nutrients and content of each products based on the reviews and comments made by citizens and stakeholders. This will ensure that ensure that the general public making key ingredients easier to see and understand by the general public.

## The Role of Biotechnology in Food Security

**Figure 2:**
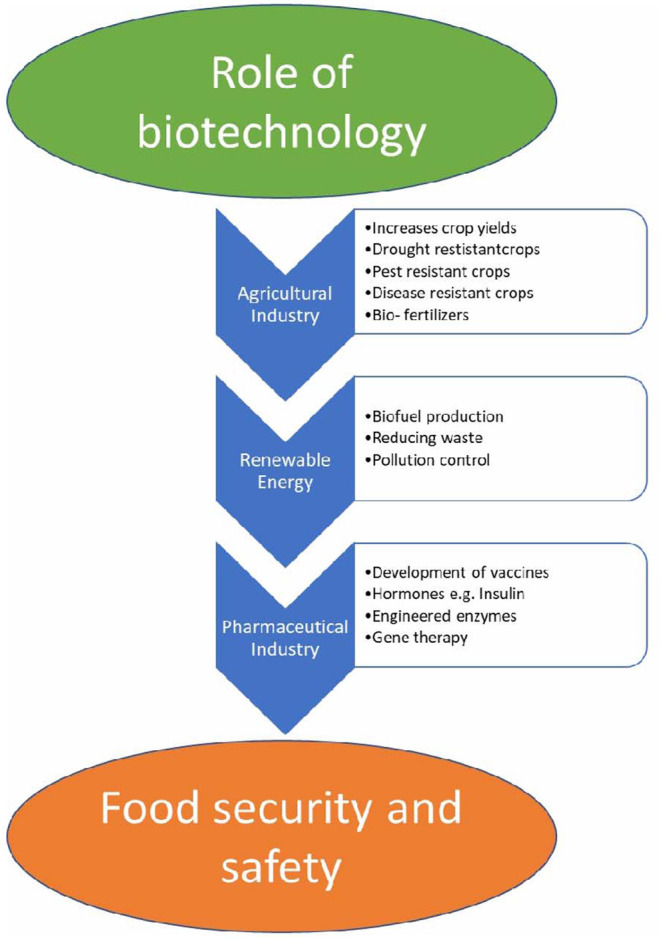
A systematic representation of the influence of biotechnology in food security and safety.

As described within this review there are several socio-environmental factors that influence food behavior in Canada these including but not limited to food insecurity, cost of foods, healthy eating initiatives, physical environments, obesogenic environments and phenomena along with governmental initiatives to address these issues through Canada's Food Guide policy and food labeling initiatives (35). It is noteworthy to point out that these above stated factors influencing food behavior are also challenges frequently experienced by many other countries around the globe and not unique to Canada alone (35). Although *prima facie* examination of many of these influencing factors may seem “unrelated” to one another in some instances, a universal way of addressing these diverse problems has been by the development and investment in the use of many readily available biotechnology strategies (63). A systematic framework representing the influence of biotechnology on food security can be shown in Figure 2. Scientific advancements in the field of biotechnology such as genetic engineering (GE) have allowed humanity to produce foods that are high in quality nutrition wise, more drought and disease resistant, and easier to produce through engineering resources that have been in use for a while (64). These technologies have made, and will continue to make, affect the food market and agriculture substantially. For instance, many of the issues related to food insecurity have been undertaken by the development and usage of biotech crops to increase crop yields on limited arable land while simultaneously addressing the concern of increasing food costs (65). In another example, the biotech development of drought and disease resistant food crops has addressed the problems stemming from global climate change while allowing dwindling available farmland to be used more efficiently (66). Other biotechnology advances have targeted social needs for more nutritious food through the introduction of many GM foods while some biotech crops have made foods healthier (more organic like) by reducing pesticide usage with the added advantage of protecting the environment from the contamination by lowering overall pesticide usage (http://www.biotech.ca/policy-matters/agriculture-industrial/).

In Canada, as well as in several countries though-out the world, many programs have been pursued to find a way to harness the power of biotechnology in bio-fuel production (providing renewable and more environmentally friendly fuels); recovery of raw materials using bioengineered bacteria instead of the use of more toxic chemicals and in crop fertilization (through incorporation of nitrogen-fixing bacteria in non-nitrogen fixing plants such as cereals) (67). In addition, biotechnology has assisted in managing waste treatment and pollution control (through biological control) and the production of several health-care products and pharmaceuticals, new feedstuffs, new sources of industrial chemicals, and pest control (https://www.thecanadianencyclopedia.ca/en/article/biotechnology). In short, biotechnology has enabled us to produce foods which meet the needs of many stakeholders from the plant breeders and farmers to the consumer and consumer groups (63). Biotechnology has also ushered in the need for more public education of the consumer as it relates to health and by so doing helps change public perception on how best to maintain health while reducing health costs in the future (63).

The use of biotechnology in providing healthy food for people satisfying various stakeholders with different but complimentary needs and requirements can best be approached as indicated above by what has been called a “Food System Approach” (68). Such a Food System Approach allows for a systematic and interdisciplinary approach which ensures that all challenges are addressed from a variety of perspectives benefiting the consumer through policy making whether conducted by business independently or governments unilaterally (68). Until today there has been no overarching global policies which govern the use of biotechnology in food production, rather there exists a patchwork of policies which aim to ensure both human and animal health at the national or regional level (69). While, each policy may approach the issue of human and animal safety in a slightly different manner, they all have been successful at permitting biotechnology to be used as a potential means of meeting the ever increasing nutritional and health challenges of both humans and animals in recent years (69).

## Conclusion

In summary, the review of literature within this paper have discussed various socio-environmental determinants affecting eating behavior of the general public in Canada. Literature has suggested that some of the main determinants affecting the eating habits include a combination of economic social and physical environments. There appears to be a significant need to address the creation and execution of policies in the food and nutrition environment. The Federal government in Canada also has a role to play in ensuring healthy eating habits among its population by putting in place policies that are in tandem with standard food and nutrition policies and a good example is setting a standard for the amount of trans fat and sodium in processed foods, guidelines for foods and beverages in school vending machines. A policy that mandates restaurants to list calorie content of food in their menu is not also out of place.

In order to improve the various types of nutritious foods and public awareness of the nutritional information, policies need to be adopted by various stakeholders. These include the food and beverage industry, media advertisements, and fast food restaurants. This will improve not only overall health but also improve the social well-being of the general public. Currently, governments such as ours in Canada are re-examining the importance of human health and relationship of agricultural food chain and agribusinesses in supporting human health.

## Author Contributions

All authors listed have made a substantial, direct and intellectual contribution to the work, and approved it for publication.

## Conflict of Interest

The authors declare that the research was conducted in the absence of any commercial or financial relationships that could be construed as a potential conflict of interest.
